# Starve to Sustain—An Ancient Syrian Landrace of Sorghum as Tool for Phosphorous Bio-Economy?

**DOI:** 10.3390/ijms22179312

**Published:** 2021-08-27

**Authors:** Adnan Kanbar, Madeleine Mirzai, Eman Abuslima, Noemi Flubacher, Rose Eghbalian, Krassimir Garbev, Britta Bergfeldt, Angela Ullrich, Hans Leibold, Elisabeth Eiche, Mario Müller, Markus Mokry, Dieter Stapf, Peter Nick

**Affiliations:** 1 Molecular Cell Biology, Botanical Institute, Karlsruhe Institute of Technology, 76131 Karlsruhe, Germany; madeleinemaria@web.de (M.M.); eman_ramadan@science.suez.edu.eg (E.A.); noemi-flubacher@gmx.de (N.F.); eghbalianrose@yahoo.com (R.E.); peter.nick@kit.edu (P.N.); 2 Institute for Technical Chemistry, Karlsruhe Institute of Technology, 76344 Eggenstein-Leopoldshafen, Germany; krassimir.garbev@kit.edu (K.G.); britta.bergfeldt@kit.edu (B.B.); angela.ullrich@kit.edu (A.U.); hans.leibold@kit.edu (H.L.); dieter.stapf@kit.edu (D.S.); 3 Institute of Applied Geosciences, Karlsruhe Institute of Technology, 76131 Karlsruhe, Germany; elisabeth.eiche@kit.edu; 4 Agricultural Technology Center (LTZ Augustenberg), 76227 Karlsruhe, Germany; mario.mueller@ltz.bwl.de (M.M.); Markus.Mokry@LTZ.bwl.de (M.M.); 5 Department of Botany, Faculty of Science, Suez Canal University, Ismailia 41522, Egypt

**Keywords:** sorghum, phosphorus, P_i_ starvation, roots, *SbPht1* genes, infrared spectra, ash

## Abstract

Phosphorus (P) is an essential macronutrient, playing a role in developmental and metabolic processes in plants. To understand the local and systemic responses of sorghum to inorganic phosphorus (P_i_) starvation and the potential of straw and ash for reutilisation in agriculture, we compared two grain (Razinieh) and sweet (Della) sorghum varieties with respect to their morpho-physiological and molecular responses. We found that P_i_ starvation increased the elongation of primary roots, the formation of lateral roots, and the accumulation of anthocyanin. In Razinieh, lateral roots were promoted to a higher extent, correlated with a higher expression of *SbPht1* phosphate transporters. Infrared spectra of straw from mature plants raised to maturity showed two prominent bands at 1371 and 2337 cm^−1^, which could be assigned to P-H(H_2_) stretching vibration in phosphine acid and phosphinothious acid, and their derivates, whose abundance correlated with phosphate uptake of the source plant and genotype (with a higher intensity in Razinieh). The ash generated from these straws stimulated the shoot elongation and root development of the rice seedlings, especially for the material derived from Razinieh raised under P_i_ starvation. In conclusion, sorghum growing on marginal lands has potential as a bio-economy alternative for mineral phosphorus recycling.

## 1. Introduction

Phosphorus (P) is an essential macronutrient, playing a role in developmental and metabolic processes in plants including energy supply (ATP), gene expression (nucleotides), and signaling (protein phosphorylation). In many agricultural and natural ecosystems, P is the limiting factor of growth [1]. Plants acquire P as inorganic orthophosphate (P_i_) ions, which are usually integrated into large and rather immobile complexes in the soil and are therefore only poorly available for the plant, posing a serious constraint on plant productivity [2]. The main source of phosphate fertilisers is P_i_-containing rocks. This practice will presumably deplete the known phosphorous mines in the course of a few decades [3]. Therefore, bio-economic approaches using P_i_ recycling from plant residues will become more important to safeguard the global P reserves. However, such strategies depend on the ability of crop plants to mobilise phosphorus from insoluble complexes, mainly with Al or Fe (in acidic soils), or to forage the irregularly distributed P_i_ from alkaline soils [4]. Thus, it is mandatory to establish new crop varieties with improved P_i_ uptake efficiency, as a prerequisite for P recycling by utilisation of residues after harvesting [5].

Sorghum (*Sorghum bicolor* L.) is an interesting candidate crop, since it has the ability to grow in P_i_-poor soil and allows for P_i_ recycling from its straw [5,6]. Sorghum is a multi-purpose economically important crop for food, fodder, bio-fuel, and other industrial uses [7]. In arid and semi-arid areas, sorghum, as a C_4_ photosynthetic plant, shows high performance with excellent biomass production within a comparatively short life span [8]. As an additional contribution to the bio-economical valorisation of sorghum, the residuals from the extraction of sugars (sweet type) or from harvesting the grains (grain type) for the production of biochar or ash were explored [5]. This approach towards P recycling shifts the mechanisms of P_i_ uptake, transport, and sequestration into focus.

Plant species that are adapted to P_i_-depleted soils have evolved a set of local and systemic (long-distance) adaptive responses to exploit otherwise inaccessible phosphates in the soil and enhance P_i_ uptake and recycling [9]. The local responses involve massive alterations in the root system architecture [10], while the systemic responses include stimulation of P_i_ transport by increasing the expression of high-affinity P_i_ transporters [11]. The reorganisation of the root system has been intensively studied for the model plant *Arabidopsis thaliana*. Here, P_i_ deficiency inhibits primary root growth while stimulating the formation of lateral roots and root hairs [12,13]. To what extent the inhibition of primary root growth can be generalised is questionable, since even within Arabidopsis, only certain ecotypes, such as Col-0, show this response to P_i_ depletion stress [13]. However, the general response, that is, to allocate more resources to the roots during P_i_ deficiency and, thus, to have a longer root system, seems to be very general. This response has also been observed for sorghum [14], maize [15], ssp. *japonica* rice [16], and an additional 14 species of monocotyledonous and dicotyledonous plants tested in a hydroponic culture system [17]. This root system remodelling allows plants to forage a greater soil volume and, thus, to maximise phosphorus uptake [18].

These local responses in the roots act in concert with systemic responses. Generally, turning on the local response by P_i_-starved plants leads to the activation of long-distance signals, in order to absorb, utilise, and transport P_i_ from either internal or external pools [19]. The major road for P_i_ uptake in plants is through symplastic transport across the plasma membrane after uptake into the root hairs, which uses P_i_/H^+^ symporters belonging to the phosphate transporter 1 (*Pht1*) gene family [20]. The Pht1 proteins localise to the plasma membrane and account for the bulk of P_i_ uptake under different ambient concentrations of P_i_ [21]. While expression of *Pht1* genes occurs mostly in the roots, in response to P_i_ deficiency, some members are also active in the flowers, leaves, and stems, indicating a role for the translocation of P_i_ inside the plant [22]. The sorghum genome harbours 11 predicted members of the *Pht1* family [11]. 

A deeper understanding of these transporters might contribute to a sustainable management of the global P reserves, through P_i_ recycling by utilisation of plant residues or ash in agriculture. Known as the oldest fertilisers in the world, combusted biomass ashes delivering minerals have been used as fertilisers from the first day of human civilisation [23]. The recycling of ashes for agricultural purposes may reduce the usage of commercial fertilisers and has the potential to solve the increasing demand of biomass production and, at the same time, the problem of ash disposal [24]. Herein, the mineral ash composition is decisive for the solubility of P [25]. Sorghum ashes contain P and other nutrients needed for plant nutrition [23]. In the present study, a grain (Razinieh) variety and a sweet (Della) variety of sorghum were used to (i) explore the root architectural adaptation to P_i_ availability at the seedling growth stage (local responses), (ii) investigate the P_i_ transport through the expression of high-affinity transporters (systemic responses), (iii) compare the effect of different P_i_ availabilities in the soil on the physicochemical characterisation of straw and ash from sorghum as a crop, and (iv) assess the effect of sorghum ash on the rice seedling growth.

## 2. Results and Discussion

Phosphorous is an important element for plant growth and physiology. It is mandatory, therefore, to comprehend how plants respond to P_i_ starvation during the vegetative growth stage, and to design strategies to breed varieties with better P_i_ uptake and use efficiency. For the current study, we took advantage of the morpho-physiological and molecular differences between a sweet (Della) and a grain (Razinieh) variety of sorghum [26,27,28,29], in order to understand how different sorghum types respond to P_i_ starvation. We analysed morpho-physiological parameters, expression of key genes, and chemical speciation of phosphorous. As a contribution towards a circular economy, we also generated ash from this plant material. This allowed assessing how the P_i_ regime and genotype of the donor material modulate the fertiliser activity, using the seedling growth of rice as a readout.

### 2.1. Phosphorus Starvation Enhances Primary Root Elongation and Inhibits Shoot Growth

**Figure 1:**
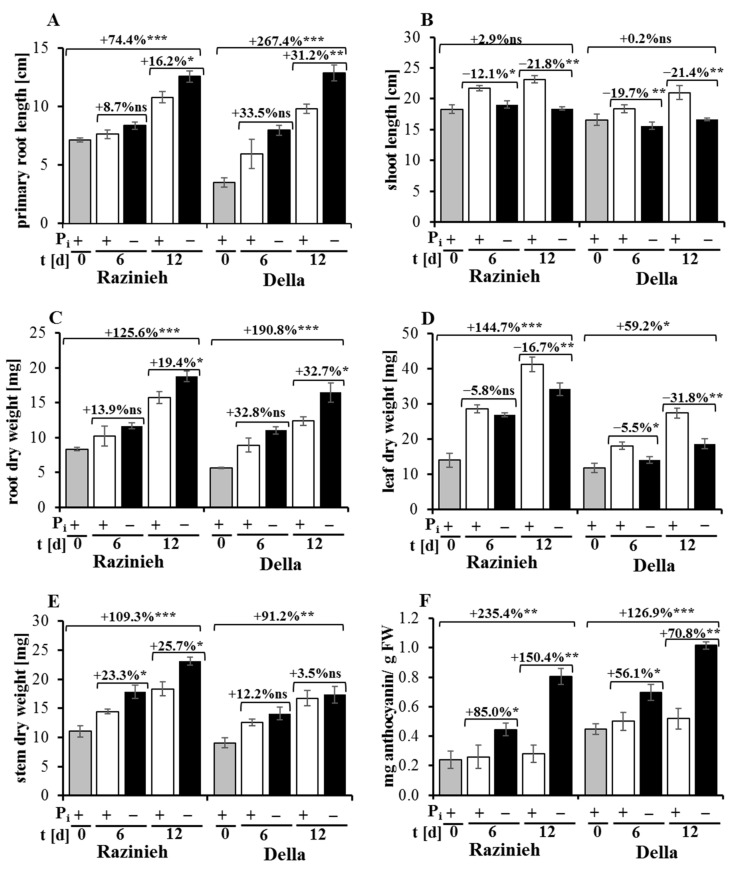
Effect of phosphorus starvation on seedling growth of a grain (Razinieh) and a sweet (Della) sorghum variety. The seeds of two varieties were initially grown in agar with 8% MS for 10 days; after that, the seedlings were transferred to half Hoagland solution for 2 days, followed by full Hoagland solution for 4 days to obtain greater adaptation. Thereafter, the seedlings were subjected to 2 mM NH_4_H_2_PO_4_ (normal condition; +P_i_) or zero (phosphorous starvation; −P_i_). After that, plant sampling was conducted at 0, 6, and 12 days of P_i_ starvation for measurement of traits. Data are presented for (**A**) primary root length (cm), (**B**) shoot length (cm), (**C**) root dry weight (mg), (**D**) leaf dry weight (mg), (**E**) stem dry weight (mg), and (**F**) anthocyanin content (mg/g FW). Values are means ± SE. ns: not significant; *, **, *** are significant at 0.05, 0.01, and 0.001, respectively, paired two-tailed Student’s *t*-test; *n* = 9.

Changing the root and shoot architecture under P_i_ starvation helps to improve the ability of a plant to forage P_i_ and represents one of the earliest phenotyping changes [30]. Therefore, we measured the response of root and shoot growth in Razinieh and Della, grown under normal conditions or under P_i_ starvation. We observed that P_i_ depletion enhanced the elongation of the seminal root, and of the root dry weight as well (Figure 1A,C). In contrast, shoot elongation stopped under P_i_ depletion (Figure 1B). Interestingly, the temporal increment in the stem dry weight, composed of leaves and the shoot axis, increased under P_i_ depletion (Figure 1E), while that of the leaf dry weight decreased (Figure 1D). Thus, the increment in the dry weight for the shoot axis increased, rather than decreased (this effect was more pronounced in Razinieh over Della). This means that the observed arrest of shoot elongation (Figure 1B) is mainly caused by arrested cell expansion (uptake of water), rather than inhibited cell proliferation. As a consequence of the stimulated root elongation and reduced shoot elongation, the ratio of root to shoot length increased as well (Supplementary Figure S1A). However, the dry weight also partitioned more towards the root because the increase in the stem dry weight in response to P_i_ depletion did not keep pace with the increase in the root dry weight (Supplementary Figure S1B). Both varieties responded to P_i_ depletion with a substantial accumulation of anthocyanin (Figure 1F). While the overall pattern was comparable between both genotypes, Della displayed a stronger reaction to P_i_ starvation, as evident from the primary root growth at day 12 of P_i_ depletion (267.4% compared to 74.4% in Razinieh). This rapid increase in the primary root length of Della was reflected by a corresponding rise in the root dry weight (190.8% compared to 125.6% in Razinieh). These differences were already manifested at day 6 of P_i_ depletion (Figure 1A,C). These morphological responses probably contribute to the adaptation to P_i_ deficiency. The extension of the root system caused by the altered cell expansion and proliferation will increase the surface available for absorption and, thus, for foraging P_i_ from a more extended area to compensate for P_i_ deficiency [15]. The stimulation of primary root elongation and root dry weight in both sweet and grain sorghum varieties under hydroponic conditions is consistent with earlier observations in sorghum [14], maize [15], and ssp. *japonica* rice [16]. The situation in Arabidopsis seems to be different, though [31], which is probably related to the completely different root system (the seminal root of *Graminea* is later replaced by a homorrhizous system, while the seminal root in dicots persists and develops into an allorrhizous system).

We observed that the shoots did not elongate under P_i_ starvation, irrespective of the genotype (Figure 1B). In addition, leaf biomass accumulated more slowly under P_i_ starvation. This inhibition of leaf growth was more prominent in Della (−31.8% at day 12 versus the control condition) compared to Razinieh (−16.7% at day 12 versus the control condition), on the background that leaf growth was, nonetheless, much slower in Della (Figure 1D). This inhibition of shoot growth at concomitantly increasing root proliferation is consistent with previous observations in rice [32], sorghum [33], and Arabidopsis [12,13]. The biological function might be that resources repartition to the root system, supporting their exploration of the soil for patches of P_i_ [18].

Anthocyanin over-pigmentation is one of the phenotypic characteristics of plants in response to P_i_ starvation [34]. Measurements of anthocyanin in the shoots (normalised by fresh weight) showed low levels for both Razinieh and Della under normal P_i_ conditions, but a remarkable increase under P_i_ depletion stress (Figure 1F). To account for the differences in leaf biomass, we normalised the anthocyanin content on the shoot fresh weight. We see that the ground level in Della is almost twice that of Razinieh, and Della also accumulated more anthocyanin under P_i_ depletion stress. However, on a relative scale, the increase in Razinieh was stronger (around 235.4% of the ground level at day 12 of P_i_ depletion) than in Della (around 126.9% of the ground level at day 12), such that under stress, the two genotypes approached each other with respect to pigmentation. These data are consistent with findings in Arabidopsis, where anthocyanin accumulation in response to P_i_ depletion does not depend on growth [35]. Although showing a reduction in leaf growth, the sweet variety Della retains anthocyanin accumulation, even at higher levels than Razinieh. This has to be seen on the background of the higher level of sugar in the shoot of Della [29] compared to Razinieh [26,27,28]. Since sugars accumulate under P_i_ starvation [36], it is straightforward to assume that glycosylation of the phenolic moiety and import to the vacuole are crucial for this pigment response. Moreover, activation of secondary metabolism means that resources have to be relocated and are not available for growth, which is a major reason why adaptation to stress comes with a growth cost [37]. However, beyond this impact as limiting factors, sugars exert regulatory functions, acting as signals and cross-talking with hormonal regulation [38]. In Arabidopsis, sucrose can activate anthocyanin biosynthesis through a sucrose importer, MAPK cascade [39]. This culminates in the induction of the transcription factor *MYB57/PAP1* that activates the genes of the anthocyanin pathway [40]. The activation of sugar signaling by P_i_ starvation is well known [41]. A straightforward working model would explain the more pronounced anthocyanin pigmentation in Della as a consequence of the more pronounced accumulation of sucrose in this sweet sorghum genotype.

In summary, the stimulation of root development (accompanied by an inhibition of shoot development) can also be used in sorghum as a reliable phenotypic marker for the adaptation to P_i_ starvation stress. In contrast, the accumulation of anthocyanin seems to depend on sugar signaling and represents a stress marker, rather than a marker for stress adaptation. Thus, Razinieh and Della contrast with respect to the adaptive versus the stress-reporting responses to Pi depletion.

### 2.2. Lateral Root Formation Is Induced by Phosphate Starvation

**Figure 2:**
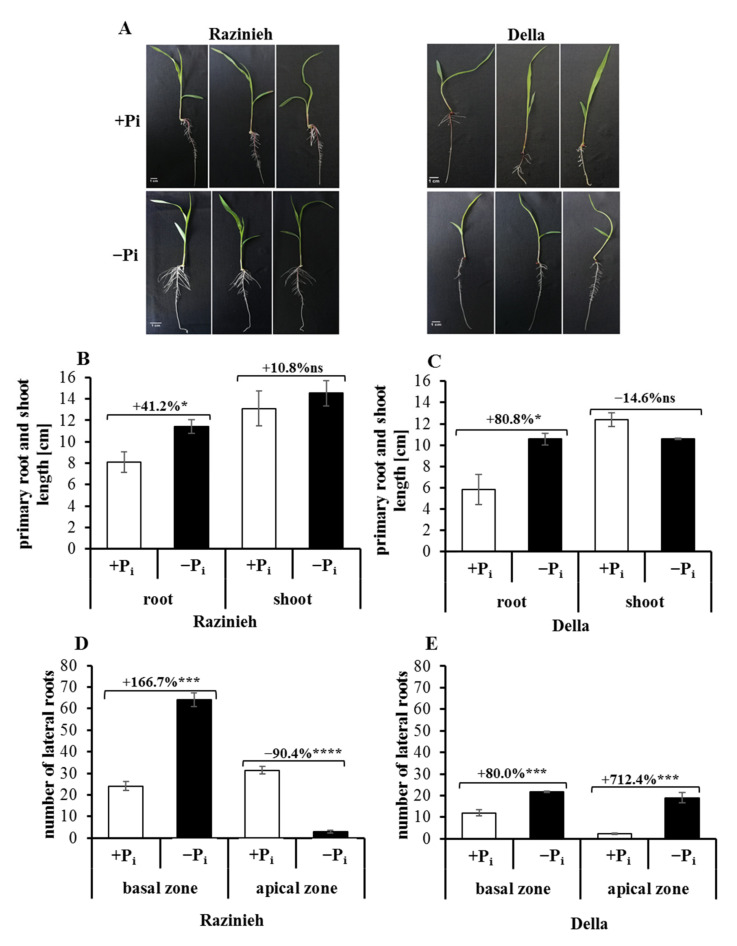
Effect of phosphorus starvation on root system architecture and growth of a grain (Razinieh) and a sweet (Della) sorghum variety. The seeds of two varieties were initially grown in agar with 8% MS for 4 days; after that, the seedlings were transferred to big plates with a wedge containing Hoagland solution and 1.5% phytoagar (pH 5.8) for 6 days. The P_i_ treatments were 2 mM NH_4_H_2_PO_4_ (normal condition; +Pi) or zero (phosphorous starvation; −P_i_). Plant sampling was conducted after 6 days of P_i_ treatments. Data are presented for (**A**) root system architecture, (**B**) primary root and shoot lengths of Razinieh (cm), (**C**) primary root and shoot lengths of Della (cm), (**D**) number of lateral roots in basal and apical zones of Razinieh, (**E**) number of lateral roots in basal and apical zones of Della. Values are means ± SE; ns: not significant; *, ***, **** are significant at 0.05, 0.001, and 0.0001, respectively, paired two-tailed Student’s t-test; *n* = 9.

Reports on the effect of P_i_ deficiency are discrepant. Even for the same model (*Arabidopsis thaliana*), both suppression [13] and enhancement [14] have been reported, and for sorghum [15], primary root growth and the production of lateral roots seem to be stimulated. Transferring the conclusions drawn from the seminal roots of Arabidopsis to sorghum is problematic, since both root systems develop through different mechanisms—the seminal root in Arabidopsis persists to give rise to a more or less allorrhizous system, while in cereals such as sorghum, the seminal root decays later and is replaced by a homorrhizous system. In our experiments, primary root elongation was stimulated by P_i_ depletion in both varieties studied (Figure 1A and Figure 2A–C), and lateral roots were promoted as well, albeit the amplitude and location differed between the two varieties (Figure 2D,E). 

Already under control conditions, lateral roots were significantly more abundant in Razinieh as compared to Della, and this difference became accentuated under P_i_ depletion. In the basal half of the seminal root, the difference in lateral root density was almost 3-fold in Razinieh over Della. Interestingly, the situation was different in the apical half of the seminal root. Here, P_i_ depletion enhanced lateral roots in Della but was inhibitory in Razinieh. As a result, Razinieh produces a much larger number of lateral roots, confined to the basal half of the seminal root (i.e., close to the soil surface). This is expected to result in a greater exploratory capacity to forage the soil for phosphorous [18].

### 2.3. Razinieh Retains More P and Fe in Roots and Shoots under P_i_ Starvation

**Figure 3:**
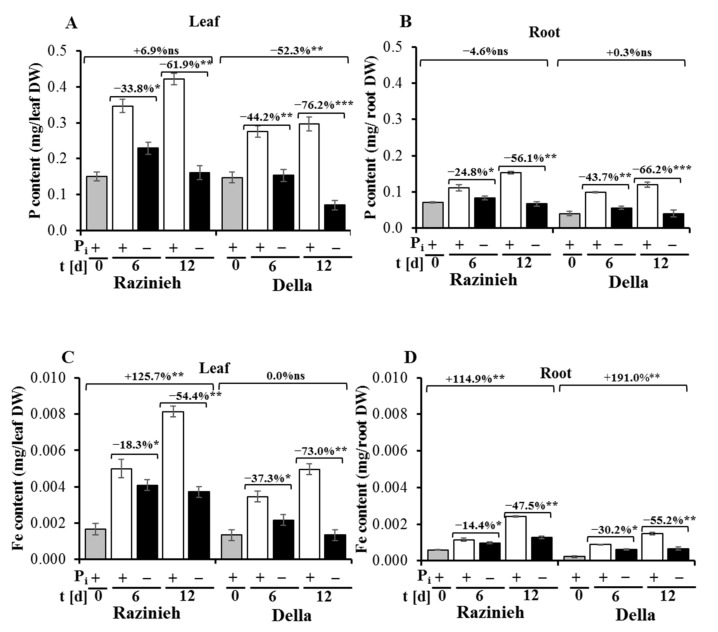
Metal nutrient analysis in the root and shoot of a grain (Razinieh) and a sweet (Della) sorghum variety grown in two varying concentrations of P_i_ hydroponically at the seedling stage. (**A**,**B**) phosphorus content (mg/g DW) in the leaves and the roots, respectively, and (**C**,**D**) iron content (mg/g DW) in the leaves and the roots, respectively. The seeds of two varieties were initially grown in agar with 8% MS for 10 days; after that, the seedlings were transferred to half Hoagland solution for 2 days, followed by full Hoagland solution for 4 days to obtain greater adaptation. Thereafter, the seedlings were subjected to 2 mM NH_4_H_2_PO_4_ (normal condition; +P_i_) or zero (phosphorous starvation; −P_i_). After that, plant sampling was conducted at 0, 6, and 12 days of P_i_ starvation for metal nutrient analysis. Values are means ± SE; ns: not significant; *, **, *** are significant at 0.05, 0.01, and 0.001, respectively, paired two-tailed Student’s *t*-test; *n* = 9.

Plants can achieve tolerance to P_i_ starvation by optimising the utilisation of P, and/or by improving P acquisition from the soil [9]. To understand whether remodeling of the root system, observed in both varieties, improved the uptake of P, we determined P and Fe contents in the leaf and root tissues of Razinieh and Della grown under both normal conditions and P_i_ depletion stress (Figure 3A,B), sampling seedlings raised under the same conditions as in Figure 1. Since the increase in dry weight was quite different between genotypes and conditions (Figure 1C,D), we calculated the P and Fe contents on the base of individual plants. As it was to be expected, the P content was significantly reduced in the leaves (Figure 3A) and roots (Figure 3B) of both varieties after 6 and 12 days of P_i_ starvation compared with the control treatment. However, the retention of P was significantly better in Razinieh as compared to Della under conditions of P_i_ depletion, which was most pronounced for the leaves. Even after 12 days of P_i_ starvation, the initial total amount of P in the leaves was maintained, while in Della, it had dropped to less than half of this value (Figure 3A). Interestingly, P_i_ depletion also resulted in a significant reduction in the Fe content in the leaves (Figure 3C) and roots (Figure 3D). Again, Razinieh was superior in retaining the total iron content under P_i_ starvation and was even able to double the initial content per root and leaf over the 12 d of the experiment, while Della was able to do so only in the roots and to a significantly lower degree.

Razinieh displayed more lateral roots, leading to a larger root surface area and root volume than Della under both normal and P_i_ stress conditions (Figure 2A). This might be one reason why this genotype was able to partially compensate P_i_ starvation (and in consequence, Fe starvation). This finding is consistent with observations on the maize line DSY2 [15] which is resistant to P_i_ depletion and also shows a greater root length, volume, and surface area as well as a higher vitality of roots, linked to a superior acidic phosphatase activity compared to the sensitive line DSY79. Overexpression of *AVP1* (Arabidopsis gene coding for a H^+^-translocating inorganic pyrophosphatase located in the vacuole) in tomato, rice, and maize induced more extended root systems that correlated with a higher tolerance to stress induced by low P availability [42,43,44]. The finding that P_i_ starvation not only impaired the accumulation of P but also of Fe (Figure 3C,D) warrants further explication. P and Fe are both essential mineral nutrients, playing crucial roles in all organisms. In the liquid phase of the soil, organic P or inorganic phosphate (P_i_) forms highly insoluble complexes with Fe or other cations, leading to a restricted phytoavailability of P_i_. Similarly, the mobility of Fe is strongly decreased by the formation of Fe oxi-hydrates and Fe phosphates that are not readily available to plants in the respective soil conditions [45]. Iron transport across plasma membranes is mediated by the Fe carrier iron-regulated transporter 1 (*IRT1*), which is highly expressed under iron deficiency. However, P_i_ starvation suppresses the expression of *IRT1* along with the low-iron-inducible ferric chelate reductase, mobilising iron in the soil [46]. This would imply that iron uptake into the seminal roots of Arabidopsis should be inhibited under P_i_ depletion. However, in contrast to this expectation, the inhibition of primary root elongation on the P_i_-deficient medium was shown not to be primarily due to the P_i_ deficiency but to toxicity by excess Fe. This might be caused by the improved mobility of Fe, which is no longer sequestered by phosphate complexes. 

As a consequence, iron accumulated in the plastids in the form of ferritin iron [47]. Thus, the activity of iron uptake was quite opposed to the pattern of uptake-related transcripts—another example for the caveat to be taken with conclusions drawn merely from transcript data. To what extent the situation can be transferred to sorghum is questionable. Not only are the root systems quite different (the seminal root of sorghum decays later, while that of Arabidopsis persists) but also root elongation is not inhibited by P_i_ depletion, but rather stimulated (Figure 1A). Just from the chemistry, P_i_ deficiency by improving Fe mobility should cause an increase in the Fe content in plant tissues. We observed just the opposite (Figure 3C,D), indicating that, unlike in Arabidopsis, P_i_ is required for efficient Fe uptake. It would be interesting to check whether the sorghum homologues of *IRT1* and *FRO2* are regulated in the same manner as in Arabidopsis or inversely (which would mean that Fe uptake is mainly regulated at the posttranslational level).

### 2.4. The Phosphate Transporter SbPT7 Is Induced under P_i_ Starvation

**Figure 4:**
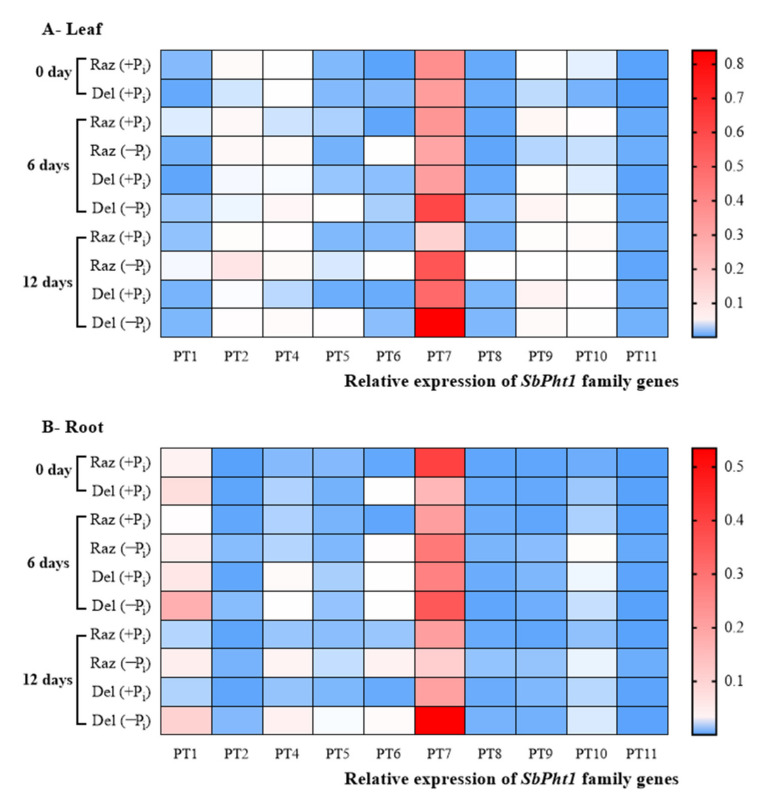
Expression profiles of 10 sorghum *SbPht1* genes in leaves and roots of a grain (Razinieh) and a sweet (Della) sorghum variety grown under P_i_ starvation treatments. (**A**,**B**) Relative expression of *SbPht1* family genes in the leaf and the root, respectively. The seeds of two varieties were initially grown in agar with 8% MS for 10 days; after that, the seedlings were transferred to half Hoagland solution for 2 days, followed by full Hoagland solution for 4 days to obtain greater adaptation. Thereafter, the seedlings were subjected to 2 mM NH_4_H_2_PO_4_ (normal condition; +P_i_) or zero (phosphorous starvation; −P_i_). After that, plant sampling for RNA extraction was conducted at 0, 6, and 12 days of P_i_ starvation. Transcript levels were normalised against the ubiquitin housekeeping gene. Values are means of three biological replicates. Raz, Razinieh; d, day. The colour scale is shown on the right side. Heat map of gene expression profiles was generated using GraphPad Prism 8.4.3 software after data normalisation.

In addition to the stimulation of root growth, P_i_ starvation might also induce the expression of P_i_ transporters of the *SbPht1* family as a mechanism to retain P homeostasis under stress. We tested this possibility by quantitative RT-PCR (Figure 4). We were able to detect transcripts for ten members of the *SbPht1* family: *SbPT1*, *SbPT2*, *SbPT4*, *SbPT5*, *SbPT6*, *SbPT7*, *SbPT8*, *SbPT9*, *SbPT10*, and *SbPT11*, while we failed to detect transcripts for *SbPT3* (Figure S2). Among those ten genes, the transcripts for *SbPT7* clearly dominated, with steady-state levels that exceeded those of the other genes by two or even three orders of magnitude. Even the next abundant transcript, *SbPT1*, was mostly found at levels of one order of magnitude lower. Thus, the pattern for *SbPT7* is clearly the most relevant. While the ground level of this transcript at day 0 was generally higher in both the leaves (Figure 4A) and roots (Figure 4B) of Razinieh as compared to Della, the response to P_i_ starvation was inversed between the two genotypes: in the roots of Della, *SbPT7* levels increased strongly and steadily, while they decreased in Razinieh. At day 12 of P_i_ starvation, *SbPT7* transcripts in Della had accumulated at five times the level seen in Razinieh. A similar pattern was seen in the leaves (Figure 4A), albeit the difference was less pronounced as compared to the roots (Figure 4B). Again, the ground levels of *SbPT7* transcripts were higher in Razinieh but decreased in response to P_i_ starvation, while they increased steadily in Della, such that at day 12 of P_i_ starvation, the transcript levels for *SbPT7* were increased by 50% over the level seen in Razinieh (which is much less than the 500% difference seen in the roots).

Interestingly, the much higher activity of the P_i_ transporter genes in Della at 6 and 12 days of P_i_ stress (Figure 4B) was not reflected in an improved accumulation of P_i_ in the roots (Figure 3B). Under P_i_ depletion, the roots of Razinieh were able to maintain a higher amount phosphorous per root compared to Della, i.e., the abundance of *SbPT7* was higher in the situation where less phosphorous was retained in the growing organ. The same holds true for the leaves (compare Figure 4A to Figure 3A). This means that the induction of steady-state levels for *SbPT7* is not able to compensate the progressive depletion of P_i_, and, thus, this induction can be considered as a marker for depletion stress rather than for adaptation to P_i_. On the other hand, the resting level of *SbPT7* prior to stress reports the ability to scavenge P_i_. Here, Razinieh is clearly superior to Della, which might explain why it is able to retain phosphorous levels under depletion (Figure 3A,B). Interestingly, the transcript levels for *SbPT7* are comparable between the shoots and roots, which means that the induction represents a systemic response. Compared to *SbPT7*, none of the other transcripts reach abundance levels worth considering for a potential relevance to stress and adaptation.

Our findings fit well with a literature report where the P content in the shoots correlated with P_i_ depletion tolerance in 29 accessions of sorghum [14], or where a haploid line tolerant to phosphate depletion was found to produce more lateral roots and to enhance P_i_ transport from the roots to shoots under P_i_ starvation [14]. The molecular mechanism behind this improved phosphate acquisition and transport seems to be the transporters of the *Pht* family. The crucial role of these transporters has been demonstrated in rice, where overexpression of *OsPht18* resulted in excessive P_i_ uptake, while a loss-of-function mutant exhibited elevated susceptibility to P_i_ depletion stress [48]. The regulatory specificity detected in the current study, where individual members are upregulated under particular conditions, was also seen in a study where sorghum and flax were co-cultivated, sharing a common mycorrhizal system [11]. Here, it was *PT11* that was expressed most abundantly, immediately followed by *PT7* (which was the dominant transporter in our study as well), while *PT3* was not expressed, again matching the pattern observed in our experiments.

In summary, the grain sorghum Razinieh shows a higher tolerance to P_i_ starvation compared to the sweet sorghum Della linked with longer roots and a higher number of lateral roots at the proximal region of the roots. This increases the root surface area and volume, leading to higher accessibility and accumulation of P_i_ in the roots and shoots. The faster local adaptation of Razinieh, manifested as more pronounced development of the proximal lateral roots and a higher ground level of the *SbPT7* transcript in the roots, is also accompanied by more efficient systemic responses, evident a higher ground level of *SbPT7* in the shoots as well and a more robust P homeostasis in the shoots under P_i_ depletion. Since the two genotypes belong to different sorghum types characterised by different sugar allocation patterns, further investigation into a potential role of sugar for low-P_i_ adaptation in sorghum represents a rewarding research topic.

### 2.5. Composition of Straw and Ash Depends on P_i_ Availability and on Genotype

Razinieh and Della samples were collected from plants grown in P_i_-depleted soil and P_i_-supplemented soil up to maturity, as described in the Materials and Methods, yielding four sets of samples. Set 1 was Razinieh grown in P_i_-depleted soil; set 2 was Della grown in P_i_-depleted soil; set 3 was Razinieh grown in P_i_-complemented soil; and set 4 was Della grown in P_i_-complemented soil. The four types of straw and ash samples were analysed to understand the effect of P_i_ depletion stress on the physicochemical characteristics of sorghum straw and the ash generated thereof. The physical and chemical properties of the two soil types showed that the soil with a low phosphate content consists of two times higher humus in comparison to the soil with a normal phosphate content (Table S1). Generally, phosphorus availability is controlled by three primary factors: soil pH, amount of organic matter, and proper placement of phosphorus fertiliser. From 20 to 80% of the total soil phosphorus is usually in organic form [49]. According to de Oliveira et al. [50], P adsorption in soils is principally positively correlated with clay and organic C, which means there can be a minor effect of the humus content on the available P content. However, the humus content in the soil with low phosphate is twice that in the soil with almost a 5-fold phosphate content. On the other hand, Yang et al. [51] revealed that there seems to be an optimum content of organic matter in soils enabling the release of maximum P. With respect to the influence of additional effects from Fe/Al and Ca/Mg concentrations, the different humus contents in the soils are supposed not to influence the available P content considerably. Therefore, while we cannot exclude an effect of the humus content, we estimate that it is relatively minor as compared to the effect of the differential P_i_ content. Moreover, the patterns observed in the hydroponic system (where the impact of humus is zero) and the soil system are congruent, which points to the same direction.

#### 2.5.1. Razinieh Accumulates and Retains P More Efficiently in the Straw

The elemental composition of the straw along with the relative abundance of the residual ash obtained from processing this straw at 550 °C is presented in Table 1. While the carbon content was very similar, independently of genotype and independently of P_i_ availability, there were significant differences with respect to the ion composition. Generally, straw from Razinieh contained more N, Cl, Na, Mg, Al, Si, P, K, Ca, Ti, Fe, and Zn compared to Della, independent of P_i_ availability. Moreover, the amount of residual ash was more than 50% higher in Razinieh as compared to Della. In addition to this dependence on genotype, P_i_ availability played a role—most elements were more abundant when P_i_ had been complemented in the soil but were scarcer under P_i_ depletion. Specifically, the P content dropped in Razinieh by around 25% when P_i_ was depleted but was still 25% higher than in Della under conditions of P_i_ supplementation. Moreover, the depletion under Pi starvation was less pronounced in Razinieh (by around 30%) compared to Della (by around 70%). Interestingly, Si contrasted with the other elements and was more than twice as abundant under P_i_ depletion as compared to Pi supplementation (again, the values were significantly higher, by a factor of >2, in Razinieh compared to Della). This deviant behaviour of Si seems highly relevant on the background that Si helps to mitigate phosphate depletion [52] and amplifies the expression of the *Pht1* phosphate transporters as well [53]. Generally, the differences between genotypes were more significant compared to the differences between P_i_ abundance within a given genotype. 

When the elemental composition of the ash obtained from the straw was analysed (Table 2), this Si pattern (mirrored also by the pattern for Ca) reflected that seen in the straw. For both elements, the contents were higher for P_i_ starvation. This increase was seen in both genotypes, but values were generally higher in Razinieh. Ash of Razinieh grown under P_i_ starvation showed significantly lower contents of S, Mg, P, and K compared to the ash from the same variety with a sufficient P_i_ content. For Della, only Cl and K showed such a decrease under P_i_ starvation. While in the straw, the content of P had been generally much higher in Razinieh (Table 1), this difference was far less visible for the ash (Table 2), indicating that a part of the P had been lost during the combustion process because it was bound to volatile compounds. Since the patterns are dependent on genotype, and since they are highly dependent on the type of element, they must be caused by biological processes that are specific.

#### 2.5.2. Quantitative Analysis of Mineral Phases in the Ash

*Crystalline phase*: The mineralogical composition of the four ashes is shown in Table 3. The main phases (>63 wt.% of the total mass) are carbonates, sylvite (KCl), arcanite (K_2_SO_4_), periclase (MgO), wollastonite (CaSiO_3_), and several phosphates. Phosphate phases account for about 9 wt.% of the phase content of the ashes. These are represented mainly by apatites besides minor amounts of NaAl(P_2_O_7_). In Razinieh, the main carbonate phase, independently of the P_i_ status during growth, is calcite (CaCO_3_), with smaller amounts of fairchildite (K_2_Ca(CO_3_)_2_) and ankerite (Ca(Fe,Mg,Mn)(CO_3_)_2_). This is also seen in Della grown under P_i_ depletion, while under P_i_ supplementation, the ash contains larger amounts of Na-Ca-carbonate fairchildite in addition to calcite and ankerite.

*Amorphous content*: The composition of the non-crystalline part of the sample is not assessable by X-ray diffraction. Therefore, the elemental composition of the amorphous content had to be determined from the difference in the chemical analysis of the bulk ash samples (Table 2) and the elemental composition calculated from the crystalline phases quantified by XRD. The content of the amorphous phase varied between 27.4 and 34.0 wt.%, which was mostly due to K (Table 3) and, to a lesser extent, to Cl and Si. The impact of K was especially pronounced in Della, with 16.3 wt.% for P_i_-complement soil (compared to only 9.5 wt.% in Razinieh), but it dropped significantly under P_i_ depletion (but with 11.3 wt.%, remaining higher than the values seen in Razinieh, although, here, P_i_ depletion reduced the value only slightly to 8.4 wt.%). Interestingly, Cl was strongly (by a factor of 4) increased under P_i_ depletion, but exclusively in Razinieh. For P, around 15 (Razinieh, P_i_ depletion) to 30% (Razinieh, P_i_ complementation) was not incorporated in the crystalline phases. 

#### 2.5.3. Physicochemical Characterisation of Mature Sorghum Straw and Ash

**Figure 5:**
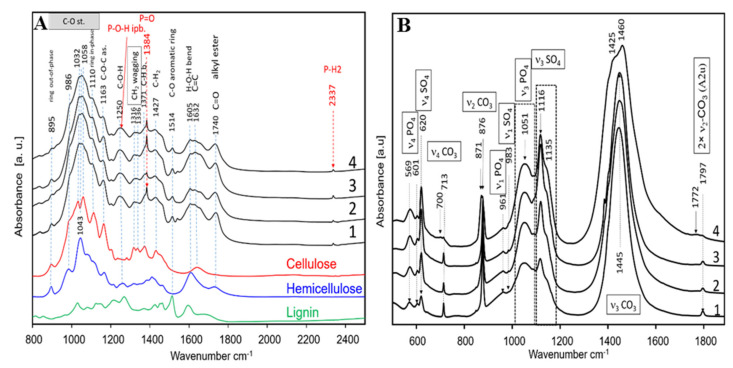
Infrared spectra of (**A**) the straw samples in the range 800–2500 cm^−1^ and (**B**) ash samples in the range 500–1900 cm^−1^. Razinieh and Della samples taken at maturity from plants grown in phosphorous-poor soil are labelled 1 and 2, respectively. Corresponding samples grown in phosphorous-rich soil are labelled 3 and 4, respectively. In addition, IR spectra of the straw organic constituents: cellulose, hemicellulose (xylan), and lignin, are shown in Figure 5A.

*IR spectroscopy of straw*: To obtain more insight into the chemical composition of the straw, we used infrared (IR) spectroscopy (Figure 5A), comparing to reference spectra of the main constituents of plant cell walls (cellulose, hemicellulose, and lignin). The main absorption bands are seen between 900 and 1200 cm^−1^ and could be attributed to C-O stretching vibrations of the polysaccharides, cellulose, and hemicellulose [54,55]. Typical ring vibrations are seen at 895 cm^−1^ (out-of-phase) and 1110 cm^−1^ (in-phase). C-O-C anti-symmetrical stretching is seen at 1163 cm^−1^. The broad bands centred at 1250 cm^−1^ report C-O-H stretching vibrations typical for lignin and hemicellulose, while CH_2_ wagging bands (cellulose) are positioned at 1316 and 1336 cm^−1^. Typical hallmarks for lignin are the C-O vibrations of aromatic rings seen at 1514 cm^−1^. Between 1550 and 1700 cm^−1^, H-O-H bending bands from hemicellulose and C=C bands are found. At 1740 cm^−1^, a band assigned to C=O stretching vibrations in the alkyl ester is observed. In general, the IR spectra of all four samples are very similar and could be attributed to combinations of the spectra of the main plant cell wall constituents.

Nevertheless, there are two great exceptions. All plants possess a sharp band at 1371 cm^−1^ with varying intensity between the samples. In addition, a sharp band with a low intensity is also seen at 2337 cm^−1^ that is not typical for the main constituents. These “exceptional” bands could be assigned to P-H(H_2_) stretching vibration in phosphine acid H_2_POH, phosphinothious acid H_2_PSH, or their derivates [56,57]. The band at 1371 cm^−1^ could be assigned to P=O vibration [58]. The frequency of P=O stretching is strongly dependent on the Cl and F derivates of phosphine oxide, leading to vibrations at 1372 cm^−1^ [58,59]. The very broad band at 1250 cm^−1^ could also be influenced by P-O-H in-plane bending vibrations, as described by Rudolph [60]. The intensity of the bands at 1371 cm^−1^ was different between the four sets—it was almost absent in Della under P_i_ depletion but appeared when Della was grown under P_i_ complementation. Instead, this band was already well pronounced in Razinieh even under P_i_ depletion and became even more prominent when P_i_ was complemented (Figure 5A). Thus, the phosphine oxide band correlated well with the quantity of phosphorous found by XRF and XRD (Table 1).

*IR spectroscopy of ashes*: When the residual ashes from the four samples were investigated (Figure 5B), the bands visible could be unambiguously assigned to carbonate, phosphate, and sulfate species as follows: The bands at 569 and 601 cm^−1^ belong to bending vibrations of type ν_4_ in the PO_4_ tetrahedra present in the apatite structure. The corresponding P-O stretching vibrations are seen as broad bands with a low intensity at 961 cm^−1^ (ν_1_ symmetrical stretching), and a very broad band centred at 1051 cm^−1^ (ν_3_ asymmetrical stretching), respectively. The sharp band at 713 cm^−1^, seen clearly in the spectra of the four samples, is due to in-plane bending (ν_4_ CO_3_) of calcite, CaCO_3_. This band in the spectrum of sample 4 (Della grown in P_i_-supplemented soil) is of a very low intensity compared to the remaining samples. An additional band at 700 cm^−1^ is present, which could be attributed to the ν_4_ CO_3_ of K_2_Ca(CO_3_)_2_ (fairchildite). The band at 876 cm^−1^ is assigned to out-of-plane bending ν_2_ of CO_3_ in calcite, and, in some cases, this vibration was split, resulting in an additional band at 871 cm^−1^, reporting the presence of fairchildite. These hallmarks of a high abundance of fairchildite at a simultaneously lower amount of CaCO_3_ were observed in Della grown in P_i_-supplemented soil, contrasting with the other three conditions. This conclusion is supported by further fingerprints such as a harmonic of the second order seen at 1797 cm^−1^. The corresponding asymmetrical stretching of the carbonate group in CaCO_3_ gives rise to a very intense band at 1445 cm^−1^ shown in both Razinieh samples and P_i_-depleted Della. Instead, P_i_-complemented Della shows an asymmetrical band at 1460 cm^−1^ and a shoulder at 1425 cm^−1^ characteristic of K_2_Ca(CO_3_)_2_. Thus, the IR data confirm the XRD results (Table 3) with respect to the high abundance of fairchildite.

A further specific difference concerned K_2_SO_4_ (arcanite), unambiguously reported by a sharp band at 1116 cm^−1^ and a shoulder at 1135 cm^−1^, characteristic of the asymmetrical S-O stretching of SO_4_ tetrahedra. Further characteristic features of arcanite were ν_4_ bending vibrations manifested as a band at 620 cm^−1^ and the symmetrical stretching vibration (ν_1_), seen as a band with a very low intensity at 983 cm^−1^ (only Raman-active). Again, these bands corresponded well to the XRD data, confirming that ash from Razinieh grown under P_i_ depletion showed the lowest arcanite level but rose to the highest level when raised in the presence of sufficient P_i_, while Della maintained intermediate levels irrespective of the P_i_ regime, which is again in line with the XRD data (Table 3).

In summary, the chemical analysis of the straw and ash generated from this straw showed that the elemental composition was dependent on both the genotype and P_i_ regime. The patterns obtained by X-ray diffraction and those measured by IR spectroscopy matched well, demonstrating the validity of the results. Again, Razinieh turned out to be more efficient in P accumulation, especially if grown in the presence of sufficient P_i_. Instead, under the same conditions, Della accumulated K, which, in the ash, became prominent as fairchildite. A prominent feature was the accumulation of Si in response to P_i_-depletion. This phenomenon was seen in both genotypes but was clearly more pronounced in Razinieh.

### 2.6. Sorghum Ash Accelerates Seedling Development in Rice

**Figure 6:**
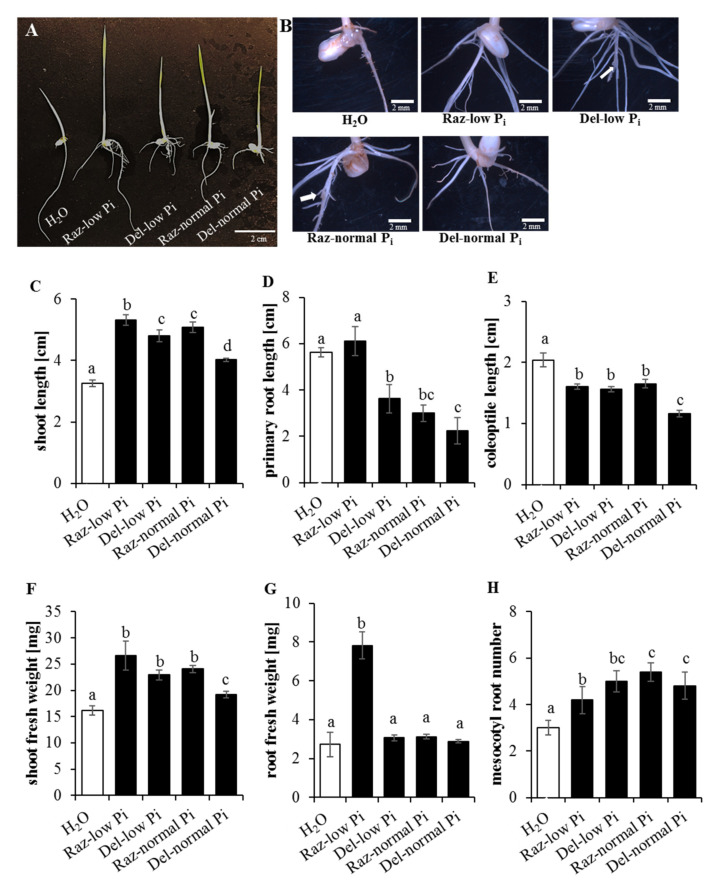
Effect of sorghum ash fertilisation on rice seedling growth. (**A**) Seedlings of Nihonmasari rice variety that have been raised for 8 d in five different hydroponic growth media: (1) H_2_O; (2) 0.5 g of Razinieh’s straw ash grown in P_i_-poor soil up to maturity dissolved in 100 mL of H_2_O, Raz-low P_i_; (3) 0.5 g of Della’s straw ash grown in P_i_-poor soil up to maturity dissolved in 100 mL of H_2_O, Del-low P_i_; (4) 0.5 g of Razinieh’s straw ash grown in P_i_-rich soil up to maturity dissolved in 100 mL of H_2_O, Raz-normal P_i_; and (5) 0.5 g of Della’s straw ash grown in P_i_-rich soil up to maturity dissolved in 100 mL of H_2_O, Del-normal P_i_. (**B**) Root growth under different ash treatments in the hydroponic growth media. (**C**–**H**) Effect of different ash treatments on the shoot length (cm), root length (cm), coleoptile length (cm), shoot fresh weight (cm), root fresh weight (mg), and mesocotyl root number. Values are means ± SDs of 5 replicates. Bars with different letters are significantly different (ANOVA, Tuckey HSD test, *p* ≤ 0.05). White arrow indicates the damage in the primary root.

The reutilisation of biomass ashes for agricultural purposes is an important approach to create nutrient cycles and reduce the usage of commercial fertilisers. One objective of this work was to demonstrate the utility of the recycling of sorghum straw grown in P_i_-depleted soil through ash production. We used the ash from Razinieh and Della shoots grown to maturity on either P_i_-depleted or P_i_-complemented soil (see above) and tested its effect on the seedling development of the *japonica* rice variety Nipponbare. In fact, we observed significant differences compared to rice seedlings raised under the same conditions on water without ash (Figure 6). Wood and plant ashes (as with sorghum) are composed of many major and minor elements needed by the plant for growth [61,62]. The motivation of the current study comes from a bio-economy approach, where sorghum (which is unique due to its sugar content, allowing the production of bio-ethanol), after being used for bio-fuel production, is further exploited for chemical compounds in the straw (lignin precursors that can be used as building blocks for bioplastics) [7], and the residue can then be used as biochar or ash for fertilising [23].

Irrespective of the genotype and P_i_ status during cultivation, there was an increased shoot length (Figure 6C) and shoot fresh weight (Figure 6F), but also an increased number of crown and adventitious roots at the mesocotylar node and the mesocotyl (Figure 6H). Instead, the length of primary roots (Figure 6D) and coleoptiles (Figure 6E) was clearly decreased. Both coleoptiles and seminal roots are primary organs that are laid down during embryogenesis and do not persist beyond the seedling stage. In fact, the development of the crown root system is suppressed by the growing coleoptile through a signal that depends on polar auxin transport [63]. When the coleoptile has completed its development, it will open at a pre-formed seam, depending on jasmonate signaling [64]. This will not only release the expansion of the embryonic leaves that were folded inside the coleoptile but also the emergence of crown roots, and, thus, the development of the secondary root system that replaces the seminal roots in all *Poaceae*, including rice and sorghum itself. Thus, the complex patterns induced by the ashes can be understood as an acceleration transition from unfolding the embryonic structures towards the architecture of the mature plant. While the overall pattern looks similar, there is one case sticking out, namely, the fresh weight of the roots, comprising both seminal and crown roots, in Razinieh grown under P_i_ starvation (Figure 6G), which is almost four times as strong as compared to the ashes from the remaining three conditions as well as that of the water control. While this is partially due to the lacking inhibition of primary root elongation in this set, contrasting with the other three sets (Figure 6D), this is not sufficient to explain the strong increase in the root fresh weight. Likewise, the number of secondary roots, while elevated compared to the control (Figure 6H), is not able to account for the observed 4-fold fresh weight of rice roots treated with the ash from P_i_-depleted Razinieh. This leads to the conclusion that the secondary root system becomes more extended under these conditions (in other words: the rice mimicked the root development of the sorghum, which was the source for this type of ash).

Since the ash is alkaline, raising the pH from 6.5 (control treatment, water) to 9.94 ± 0.10 (ash treated, no significant difference between donor genotypes and their P_i_ regimes during cultivation), it is important to ask whether the observed changes might be mere consequences of alkalinity stress. In fact, alkalinity caused damage to the root system of rice [65], albeit the observed changes in the fresh weight were much lower than those found in our study. Moreover, the induction of alkalinity stress by sodium carbonate is a composed type of stress containing an osmotic and an ionic (sodium) component as well. In a comparative study, where these components were dissected in rice [66], the inhibition of root growth was much more drastic. However, it was not accompanied by a stimulation of shoot development as in the ash-treated rice seedlings, but by a clear inhibition. Furthermore, the stimulation of the secondary root system is difficult to reconcile with a hypothesis where the effects of the ash can be attributed to alkalinity stress. A third argument is the marked differences in the pattern using ash from P_i_-depleted Razinieh (although the pH induced by this ash was the same as in the other sets). Thus, while the increase in pH does not come as a surprise given the high calcium content (11.7 to 18.9% of the dry matter, see Table 2), and while alkalinity can modulate plant growth negatively [67] also in rice [66,68], alkalinity cannot account for the observed acceleration of secondary development (secondary root system, increase in leaf development). 

The major amount of P in the ash of the four investigated samples is incorporated in different apatite structures (Cl, F, OH). Apatites are not water-soluble but require HNO_3_ or acids with pH < 5.5 [69] to be dissolved. Neither calcite nor ankerite shows solubility in water. Of the carbonate minerals, only fairchildite is soluble. The Na-Ca-carbonate reacts with water to form buetschliite, a hydrous potassium calcium carbonate (3K_2_CO_3_·2CaCO_3_·6H_2_O) and calcite (CaCO_3_). Further addition of water leads to the decomposition of buetschliite to calcite by removal of the water-soluble potassium carbonate [70]. Wollastonite and periclase are insoluble in water as well [71]. The highest solubilities were found for sylvite (347 g/L) and for arcanite (111.5 g/L). The reactions from KCl and K_2_SO_4_ are expected to acidify the medium, which should improve the solubility of phosphates. Still, the high (9.94) pH of all our ash preparations implies that the effect of the differential P content found in the ash should be low under these conditions. This might be different in soils where organic acids support the release of insoluble minerals by acidification, chelation, and exchange reactions [72]. While the massive stimulation of secondary root development observed for ash from P_i_-depleted Razinieh (Figure 6G) is unlikely to be caused by a higher release of P (in fact, the ash of P_i_-complemented Razinieh is richer in P, Table 2, but does not yield a comparable stimulation of root growth), it is well correlated with the content of Si. This element not only supports the cell wall stability and resilience of plants against pathogens and herbivorous insects [73] but also supports resilience against drought stress in sorghum [74]. Interestingly, specifically in *Poaceae*, Si also stimulates the development of the Casparian strip in the root endodermis, which contributes to maturation and the tightness of ionic homeostasis [75]. The beneficial effect of Si under P_i_ starvation is a phenomenon that has been known for a long time [76,77] and seems to be linked with an improved iron homeostasis, leading to a better bioavailability of P, at least in rice [78]. The beneficial effect of Si on P uptake was shown to be linked with an upregulation of *Pht1* transporters in wheat [53]. Uptake of Si itself is driven by specific membrane transporters [73]. A straightforward explanation for our results would be that Razinieh is endowed with a higher abundance of Si importers or with a higher activity of these importers, which would then stimulate the expression of *Pht1*. This would explain why the steady-state levels of *PT7* are highly elevated in Razinieh from the very beginning (Figure 4B), and why the silicon content is more elevated in Razinieh. An open question would be whether Si importers, such as *OsLsi1*, are downregulated by P_i_. In conclusion, potential improvement in P uptake by Si from the water-insoluble apatite mineral needs further investigation.

## 3. Materials and Methods

### 3.1. Plant Material

This study made use of a grain sorghum (*Sorghum bicolor* L. cv ‘Razinieh’) variety, and a sweet sorghum (*Sorghum bicolor* L. cv ‘Della’) variety. Razinieh is an improved Syrian landrace [26,27,28], while Della is a sweet sorghum variety, developed from a cross of Dale and ATx622 by Bob Harrison, Virginia Polytechnic Institute, and released in December 1991 [79].

### 3.2. Hydroponic Culture of Sorghum under P_i_ Starvation

To control the P_i_ availability, we developed a hydroponic strategy using seedlings. For surface sterilisation, we treated the seeds in 70% ethanol for 60 s and subsequently washed them with double-distilled water. Then, we transferred the seeds to 5% sodium hypochlorite for 20 min, followed by three washing steps in sterilised double-distilled water. The seeds were sown on 0.5% phytoagar medium (Duchefa, the Netherlands) mixed with 8% Murashige–Skoog salts, and incubated for 10 days in a climate chamber at 25 °C at a light intensity of 120 μmol m^–2^s^–1^ photosynthetically available radiation, and a cycle of 12 h light and 12 h dark. Subsequently, the seedlings were transferred to sterilised racks floating on 0.5× Hoagland solution (Supplementary Table S2) for 2 days, prior to transfer to full-strength Hoagland solution for 4 days to allow the plants to adapt to the new environment before starting the actual experiment. To follow the responses to P_i_, we split the seedlings that were now 16 d of age into two sets. The control set was growing on standard Hoagland medium with a normal supply of P_i_ (+P_i_; 2mM P_i_), while the P_i_-deficient set (−P_i_, 0 mM P_i_) developed on a modified Hoagland medium omitting the usual 2 mM NH_4_H_2_PO_4_ and adding, instead, 2 mM KCl (Supplementary Table S2). In our hydroponic system, there was only one compound containing P_i_ (NH_4_H_2_PO_4_). Thus, the required concentration of P_i_ (2 mM) in the final solution was adjusted based on the concentration of ammonium dihydrogen phosphate in the stock solution. All the glassware as well as the H_2_O used in the experiment was free of any P_i_. We checked and adjusted the pH to 6.5 on a daily basis using 0.5 M HCl and replaced the solution every second day. Otherwise, the cultivation conditions were as described above. Each data point represents 3 biological replicates.

At the onset of the experiment, and at days 6 and 12 of treatment, we scored the following morphological parameters: primary root length (cm), shoot length (cm), root dry weight (mg), leaf dry weight (mg), and stem dry weight (mg). In a further step, the ratios of root to shoot length and weight were calculated. To measure anthocyanin content [80], we sampled aliquots of around 100 to 150 milligrams of leaf fresh weight. The remaining material was used for the determination of ion content. For this purpose, we collected leaves and roots separately for each individual replicate and dried them at 70 °C for 72 h before determining P and Fe contents by atomic flame spectrometry (AAnalyst^TM^ 200, Perkin-Elmer 3030 B, Waltham, MA, USA) from 50 mg of dry weight according to Hazman et al. [81].

### 3.3. Measuring Lateral Root Growth under P_i_ Starvation

The seeds of Della and Razinieh were surface sterilised as described above and sown on plain phytoagar (0.5% *w/v*) without MS medium. After synchronisation of germination for 4 days in a climate chamber under the same conditions as in the previous experiment, we transferred the seedlings into custom-made root observation containers containing Hoagland solid medium. These containers consisted of two glass plates (21 by 30 cm) separated by 0.5 cm plastic strips. The space between the two layers was filled with either standard (2 mM P_i_; +P_i_) or P_i_-deficient (0 mM P_i_; −P_i_) Hoagland medium with 1.5% phytoagar at pH 5.8. The filled containers remained horizontally for 24 h until full solidification, before insertion of the seedlings and transfer back to the climate chamber, where the containers stood vertically, such that the roots were growing downwards, in parallel to the plates, and thus were accessible to measurement. To prevent light from affecting root growth, we wrapped the containers thoroughly with aluminum foil. We assessed root length (cm), shoot length (cm), and number of lateral roots in the apical and the basal half of the seminal roots after an additional 6 days for both Della and Razinieh. Data represent three independent experimental series.

### 3.4. Quantification of SbPht1 Transcripts

To study the expression of *SbPht1* family genes in the leaves and roots of Razinieh and Della in response to P_i_ starvation treatments, we raised seedlings as described in the first experiment (3.2) either in the presence of P_i_ or under depletion of P_i_. We sampled the roots and the second leaf at days 0, 6, and 12 after the start of the differential P_i_ treatment, immediately froze them in liquid nitrogen, and stored the samples at −80 °C until further processing. Total RNA was isolated using the Spectrum™ Plant Total RNA Kit (Sigma, Taufkirchen, Germany) according to the instructions of the manufacturer from a small amount of tissue ground to a powder (Tissue Lyzer, Qiagen, Hilden, Germany). The extracted RNA was reverse transcribed into cDNA by *M-MuLV Reverse Transcriptase* (New England Biolabs, Frankfurt am Main, Germany) using 1 μg of total RNA as a template. Real-time qPCR was performed with the CFX96 Touch™ Real-Time PCR Detection System from Bio-Rad Laboratories GmbH (Munich, Germany) using an SYBR Green dye protocol according to Svyatyna et al. [82]. Transcript levels were compared between the different samples using the 2^−ΔCt^ method [83] against *SbUBI* as a reference gene. Data represent three biological replicates (in technical triplicates) consisting of three individual plants for each condition and replicate. For details on the primers for *SbUBI* and the *SbPht1* gene-specific primers according to Walder et al. [11], refer to Table S3. To generate a heat map of steady-state transcript levels, we used the GraphPad Prism 8.4.3 software (San Diego, CA, USA) based on the 2^-ΔCt^ values after normalisation against the housekeeping gene *SbUBI*.

### 3.5. Chemical and Mineralogical Analysis of Sorghum Straw and Ash from P_i_-Rich and -Poor Soils

To simulate the situation in the field, we raised Della and Razinieh from sterilised seeds in 20 L pots filled with soil in a completely randomised design with three biological replicates. Soil samples were collected from different regions of Baden-Württemberg, Germany, for quantification of P_i_. The soil with the lowest P_i_ content (4 mg/100 g) was defined as P_i_-depleted soil, comparing to average soil (19 mg P_i_/100 g), and this average soil was supplemented with 40 mg P_i_/100 g by chemical fertilisation (calcium dihydrogen phosphate). The soil with the high P_i_ content (40 mg/100 g) was defined as P_i_-rich soil. The chemical composition of the soil was analysed regularly after each addition of fertiliser to assess the concentration of P_i_. For details on the soil structure, pH, and contents of potassium, magnesium, and phosphorus in these soils, refer to Table S1. Plants were supplemented on a daily basis during the entire growing period with de-ionised water to maintain a water capacity of 65%. The experiment took place under outdoor conditions, but with bird protection by a wire cage. Each pot received, over the growth period, 8.0 g nitrogen (as ammonium nitrate), 6.0 g potassium (as potassium chloride), and 2.5 g magnesium (as magnesium sulfate heptahydrate) in four equal portions. The experiment yielded four types of samples: (1) Razinieh in P_i_-poor soil, (2) Della in P_i_-poor soil, (3) Razinieh in P_i_-rich soil, and (4) Della in P_i_-rich soil. At maturity, we harvested the plants for analysis by X-ray diffraction, infrared spectroscopy, and chemical analysis.

#### 3.5.1. Chemical Analysis

We cut and homogenised the dried straw samples and split each sample into two parts. One part served as a specimen for chemical analysis of the straw, and the other part went into ashing according to DIN EN 14775 at 550 °C. We determined bulk concentrations of carbon, hydrogen, nitrogen, and sulfur according to DIN EN 15104 (Leco Truspec Micro, Mönchengladbach, Germany) after calibration for the concentration range present in the samples. In short, we combusted the straw samples in oxygen and subsequently analysed CO_2_, H_2_O, and SO_2_ in the combustion gases via infrared spectroscopy while determining nitrogen content by thermal conductivity measurements. We used the same method to measure the concentrations of carbon and sulfur in the plant ashes.

We measured total chlorine content in the four straws and their ash samples according to DIN EN ISO 16994, a method using combustion in oxygen in a closed vessel followed by ion chromatography (IC) analysis according to DIN EN ISO 16994. To address Na, Mg, Al, P, Si, K, Ca, Ti, Fe, Zn, and Pb, we digested the straw samples in nitric acid (9 mL) and hydrofluoric acid (1 mL) in a pressure digestion vessel, DAB-2 (Berghof). After complexation of the hydrofluoric acid with boric acid, we determined the elements via ICP-OES (iCAP 7600, from Thermo Fisher Scientific, Waltham, MA, USA) using Sc as an internal standard.

In the ashes, we quantified the same elements using X-ray fluorescence (XRF) analysis (Bruker AXS S8 Tiger, Karlsruhe, Germany) (DIN 51729-10). We prepared two beads in parallel using either 200 mg or 400 mg sample material with 6 g Li-borate salt mixtures in a Claisse M4 fluxer (Malvern Panalytical, Almelo, The Netherlands). Calibration beads were prepared accordingly. The elements Na, Al, Ti, and Pb were below the detection limit (<0.1% *w*/*w*), and therefore they are not listed in the results.

#### 3.5.2. X-ray Diffraction

We performed X-ray diffraction on the ashes using an Empyrean diffractometer (Malvern-PANalytical, Almelo, Netherlands) equipped with a multistrip PIXcel^3D^ detector (255 channels, simultaneously covering 3.347° 2θ) and Cu-radiation, filtering CuK_β_ with an HD Bragg-Brentano optics, and conducting the measurements with slits of 0.125° and Soller slits of 0.04 rad (2.3°) in the range 5–120° 2θ. For phase identification, we used the software packages Highscore-Plus V. 4.9 (PANalytical) and Diffrac-Plus (Bruker-AXS, Karlsruhe, Germany), employing the PDF 2004 (ICDD) and COD 2019 databases. For quantitative phase analyses of the four ashes, we mixed the samples with an internal standard (20 % *w*/*v* α-Al_2_O_3_) and applied the Rietveld method following the fundamental parameters approach implemented in TOPAS V4.2 (Bruker AXS, Karlsruhe, Germany), in order to account for the content of amorphous material.

#### 3.5.3. IR Spectroscopy

We analysed KBr pellets (after mixing 200 mg KBr with 0.5 mg sample) by IR spectroscopy on a Tensor II spectrometer (Bruker Optics, Ettlingen, Germany) equipped with a GLOBAR source and DTGS detector. IR spectra were collected in the range 400–4000 cm^−1^ with a spectral resolution of 4 cm^−1^. We assigned bands according to [84,85].

### 3.6. Sorghum Ash Fertilisation Effects on the Growth of Rice Seedlings

We tested potential fertilisation effects of the sorghum ash using the rice variety Nipponbare (*Oryza sativa* L. *japonica*) as a recipient. The seedlings were raised according to [81]. In short, caryopses were dehusked and surface sterilised, prior to sowing on a rubber mesh floating on the respective medium inside a magenta box. As a medium, we dissolved 0.5 g of one of the four types of sorghum ash (see experiment 1.5) in 100 mL of double-distilled water. The negative control consisted of ddH_2_O water without adding any type of ash. The seedlings developed in continuous darkness for 5 days at 25 °C in a climate chamber. Subsequently, we transferred the etiolated seedlings for an additional 3 days to a light cycle of 12 h light and 12 h dark. Then, the length of the seminal root (cm), shoot length (cm), coleoptile length (cm), shoot fresh weight (mg), root fresh weight (mg), and number of crown roots were determined as readouts for seedling development.

### 3.7. Statistical Analyses

Differences between the two sorghum varieties and the two P_i_ treatments were probed by a two-tailed Student’s t-test with a 95% confidence level using the commands PROC GLM, PROC MEANS, and PROC TTEST of the SAS v9.4 software (SAS Institute Inc., Cary, NC, USA).

## 4. Conclusions

Phosphate is an essential and limiting macronutrient, relevant for a variety of developmental and metabolic processes in plants. The uptake is mainly acquired as inorganic orthophosphate, which is poorly available in the soil due to the formation of immobile complexes. By complex adaptive local and systemic responses such as enhanced primary root elongation, increasing lateral root numbers and growth, and anthocyanin accumulation, sorghum varieties Razinieh and Della cope with P_i_ limitations. Interestingly, differences in the expression of *SbPht1* family genes were observed between both varieties, indicating the relevance of the genotype and growth conditions for the production of biomass. Thereby, the expression of *SbPht1* genes was higher in the shoots of Della and roots of Razinieh, which also exhibited more and longer lateral roots than Della during P_i_ starvation. Besides the impact on biomass production, the elemental composition was also observed to be dependent on the genotype and environment. Infrared spectra of straw grown in P_i_-rich and -poor soils showed two exceptional bands of P-H(H_2_) stretching vibration in phosphine acid and phosphinothious acid, with higher intensities in the straw of Razinieh than that of Della. Fertilisation with sorghum ash of both genotypes enhanced the shoot elongation and root number formation of rice seedlings, but ashes from Razinieh grown under P_i_ starvation 4-fold increased the root fresh weight of rice seedlings.

## Figures and Tables

**Table 1:** Chemical composition of the straw of a grain (Razinieh) and a sweet (Della) sorghum variety grown under normal levels of P_i_ or under P_i_ starvation along with the abundance of residual ash generated from this straw by processing at 550 °C.

	Razinieh		Della	
	Normal P_i_	Low P_i_	Normal P_i_	Low P_i_
Ash content at 550 °C (%)	6.46 ± 0.24	5.93 ± 0.15	4.11 ± 0.17	3.47 ± 0.04
C (%)	43.10 ± 0.52	43.20 ± 0.39	43.90 ± 0.36	44.40 ± 0.52
H (%)	5.63 ± 0.10	5.76 ± 0.03	5.77 ± 0.08	5.78 ± 0.04
N (%)	1.76 ± 0.02	1.25 ± 0.07	1.11 ± 0.01	0.99 ± 0.02
S (%)	0.29 ± 0.05	0.18 ± 0.03	0.17 ± 0.05	0.19 ± 0.05
Cl (%)	1.01	0.53	0.61	0.51
Na (mg/kg)	76.00 ± 0.58	30.0 ± 3.21	23.00 ± 0.58	14.00 ± 3.06
Mg (mg/kg)	3580 ± 10.0	2530 ± 15.3	1850 ± 10.0	1900 ± 17.3
Al (mg/kg)	98.00 ± 1.73	125.00 ± 0.71	41.00 ± 4.04	34.00 ± 0.71
Si (mg/kg)	1600 ± 10.0	3890 ± 14.1	707 ± 10.4	1580 ± 14.1
P (mg/kg)	1710 ± 5.77	1300 ± 5.77	924 ± 7.23	772 ± 16.0
K (mg/kg)	16100 ± 100	9210 ± 68.1	13000 ± 57.7	7990 ± 92.4
Ca (mg/kg)	9720 ± 20.8	11000 ± 57.7	4720 ± 17.3	5050 ± 78.1
Ti (mg/kg)	7.00 ± 0.58	7.00 ± 0.58	<4.00	<4.00
Fe (mg/kg)	109 ± 3.46	96.00 ± 1.73	55 ± 0.71	44.0 ± 1.00
Zn (mg/kg)	54.00 ± 0.00	78.00 ± 0.71	44.00 ± 1.00	37.00 ± 0.58
Pb (mg/kg)	<4.00	<4.00	<4.00	<4.00

Data in wt% or mg/kg dry matter; normal P_i_: plants grown in P_i_-supplemented soil (40 mg/100 g) up to maturity; low P_i_: plants grown in Pi-depleted soil (4 mg/100 g) up to maturity. (For Cl, only 1 analysis was possible due to the small sample amount.) Values are means ± standard deviations.

**Table 2:** Chemical analysis of plant ashes (produced at 550 °C) obtained from the straw of a grain (Razinieh) and a sweet (Della) sorghum variety grown under P_i_ starvation treatments.

	Razinieh		Della	
	Normal-P_i_	Low-P_i_	Normal-P_i_	Low-P_i_
C (%)	3.36 ± 0.07	4.61 ± 0.04	4.17 ± 0.03	4.09 ± 0.16
S (%)	2.16 ± 0.28	1.11 ± 0.16	1.59 ± 0.24	1.48 ± 0.17
Cl (%)	10.70	10.90	9.62	8.25
Mg (%)	5.49 ± 0.01	4.23 ± 0.01	4.50 ± 0.02	5.55 ± 0.00
Si (%)	2.14 ± 0.02	6.54 ± 0.04	1.68 ± 0.02	4.44 ± 0.00
P (%)	2.63 ± 0.00	2.12 ± 0.00	2.24 ± 0.03	2.20 ± 0.00
K (%)	25.60 ± 0.08	15.80 ± 0.07	32.70 ± 0.40	24.20 ± 0.00
Ca (%)	14.70 ± 0.01	18.90 ± 0.08	11.70 ± 0.01	14.90 ± 0.01
Fe (%)	0.16 ± 0.00	0.16 ± 0.00	0.13 ± 0.00	0.13 ± 0.00
Zn (%)	<0.20	<0.20	<0.20	<0.20

Data in wt% or mg/kg dry matter; normal Pi: plants grown in Pi-supplemented soil (40 mg/100 g) up to maturity; low Pi: plants grown in Pi-depleted soil (4 mg/100 g) up to maturity. (For Cl, only 1 analysis was possible due to the small sample amount.) Values are means ± standard deviations.

**Table 3:** Rietveld refinement data of XRD for plant ashes (produced at 550 °C) obtained from the straw of a grain (Razinieh) and a sweet (Della) sorghum variety grown under P_i_ starvation treatments.

Phase	Formula	Razinieh		Della	
		Normal-P_i_	Low-P_i_	Normal-P_i_	Low-P_i_
Amorphous		27.40 ± 0.8	31.80 ± 0.9	31.20 ± 1.0	34.00 ± 0.8
Hydroxylapatite	Ca_5_(PO_4_)_3_OH	6.90 ± 0.19	5.60 ± 0.2	4.00 ± 0.2	6.26 ± 0.18
Chlorapatite	Ca_5_(PO_4_)_3_Cl	1.69 ± 0.19	2.12 ± 0.18	5.40 ± 0.3	1.66 ± 0.18
Apatite	Ca_5_(PO_4_)_3_F	0.31 ± 0.05	0.22 ± 0.05	0.22 ± 0.05	0.25 ± 0.04
Na-Al-Phosphate	NaAl(P_2_O_7_)	0.30 ± 0.11	1.30 ± 0.2	0.30 ± 0.12	0.10 ± 0.10
Calcite	CaCO_3_	14.70 ± 0.14	25.50 ± 0.2	5.03 ± 0.15	13.56 ± 0.15
Fairchildite	K_2_Ca(CO_3_)_2_	5.18 ± 0.10	2.43 ± 0.11	15.87 ± 0.17	7.25 ± 0.10
Ankerite	CaFe(CO_3_)_2_	1.82 ± 0.15	3.00 ± 0.2	2.33 ± 0.17	1.77 ± 0.16
Sylvite	KCl	18.35 ± 0.16	8.61 ± 0.11	16.40 ± 0.2	14.11 ± 0.14
Arcanite	K_2_(SO_4_)	10.53 ± 0.12	4.70 ± 0.16	5.79 ± 0.13	6.81 ± 0.12
Periclase	MgO	7.57 ± 0.11	4.45 ± 0.11	7.10 ± 0.13	7.77 ± 0.12
Wollastonite	CaSiO_3_	2.26 ± 0.13	6.10 ± 0.2	3.39 ± 0.14	3.54 ± 0.15
Quartz	SiO_2_	0.71 ± 0.04	1.16 ± 0.05	0.38 ± 0.06	0.39 ± 0.04
Huntite	CaMg_3_(CO_3_)_4_	1.30 ± 0.2	0.28 ± 0.11	1.20 ± 0.2	1.40 ± 0.2
Mg-Sulphate	MgSO_4_	0.13 ± 0.04	0.83 ± 0.09	0.20 ± 0.06	0.20 ± 0.05
Halite	NaCl	0.37 ± 0.05	0.61 ± 0.05	0.44 ± 0.06	0.49 ± 0.05
Ilmenite	FeTiO_3_	0.29 ± 0.05	0.63 ± 0.08	0.23 ± 0.05	0.20 ± 0.05
Cristobalite low	SiO_2_	0.03 ± 0.02	0.17 ± 0.04	0.042 ± 0.02	0.04 ± 0.02
Rutile	TiO_2_	0.16 ± 0.06	0.49 ± 0.06	0.37 ± 0.07	0.25 ± 0.06
**Elemental composition of the amorphous phase**					
wt% of bulk sample	K	9.53 ± 0.13	8.40 ± 0.12	16.25 ± 0.42	11.33 ± 0.10
	Cl	1.60 ± 0.08	6.33 ± 0.06	1.19 ± 0.10	1.13 ± 0.07
	Si	1.24 ± 0.04	4.44 ± 0.07	0.67 ± 0.05	3.38 ± 0.04
	Ca	3.18 ± 0.14	2.44 ± 0.18	1.51 ± 0.17	3.31 ± 0.14
	C	0.71 ± 0.08	0.93 ± 0.05	1.54 ± 0.05	1.34 ± 0.16
	Mg	0.55 ± 0.08	1.19 ± 0.07	0	0.46 ± 0.08
	P	0.91 ± 0.06	0.31 ± 0.07	0.41 ± 0.08	0.67 ± 0.05

Data in wt% or mg/kg dry matter; normal P_i_: plants grown in P_i_-supplemented soil (40 mg/100 g) up to maturity; low P_i_: plants grown in P_i_-depleted soil (4 mg/100 g) up to maturity. Phase contents in wt%. Errors are esds of the refinement. Calculated elemental composition of the amorphous content given as wt% of the bulk sample. The errors were calculated from the errors of the Rietveld refinements and the errors of the chemical analyses according to Gaussian error propagation.

## Data Availability

The data presented in this study are available on request from the corresponding author.

## Supplementary Materials

The following are available online at https://www.mdpi.com/article/10.3390/ijms22179312/s1.
