# Supplementary material for: Starve to Sustain—An Ancient Syrian Landrace of Sorghum as Tool for Phosphorous Bio-Economy?
Source: Int J Mol Sci. 2021 Aug 27;22(17):9312. doi: 10.3390/ijms22179312 (PMC8430806; doi:10.3390/ijms22179312)
Supplement: Supplementary file 1 [file ijms-22-09312-s001.zip › ijms-1340933-supplementary.pdf]

Supplementary materials

Starve to Sustain – An Ancient Syrian Landrace of Sorghum as Tool for Phosphorous Bioeconomy?

Kanbar et al. 2021

List of Supplemental Figures/Tables

Figure S1. Effect of phosphorus starvation on root/shoot partitioning of a grain (Razinieh) and a sweet (Della) sorghum variety.

Figure S2. Schematic models of the upregulated *SbPht1* genes family in leaves and roots of a grain (Razinieh) and a sweet (Della) sorghum varieties grown under P_i starvation treatments.

Table S1: Physical and chemical properties of soil.

Table S2: Chemical composition of modified Hoagland's solution.

Table S3: Primers for qRT PCR analysis of *Pht1* transcripts of sorghum.

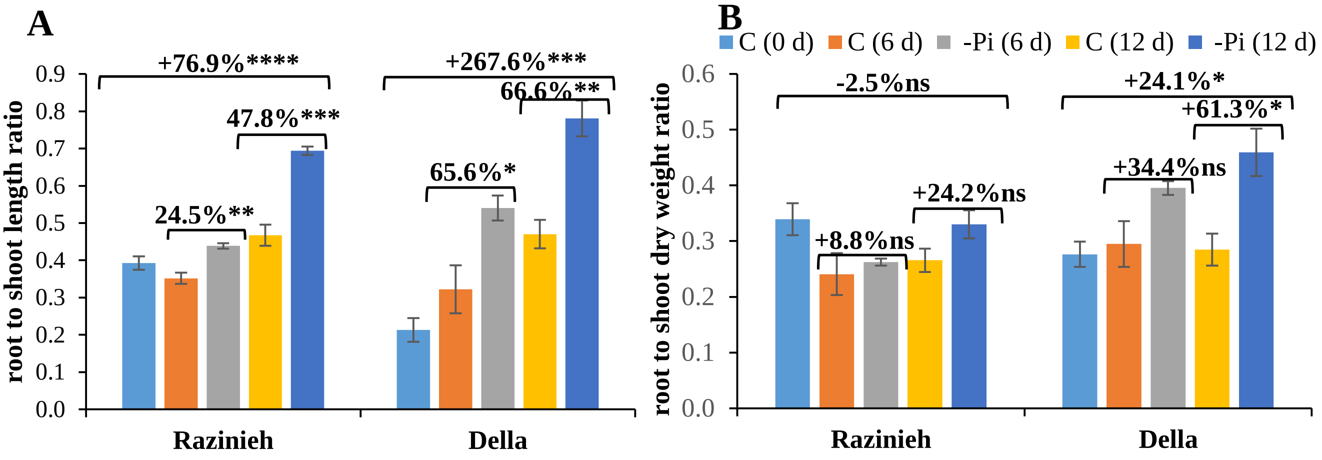

Figure S1. Effect of phosphorus starvation on root/shoot partitioning of a grain (Razinieh) and a sweet (Della) sorghum variety. Seedlings were raised either under control conditions (C), or under P_i depletion as described in material and methods. Ratios of organ lengths are given in (**A**), ratios of dry weight in (**B**). Values are means±SE; ns: not significant; *, **, ***, **** are significant at 0.05, 0.01, 0.001, 0.0001, respectively. paired two-tailed Student's t-test; n=9.

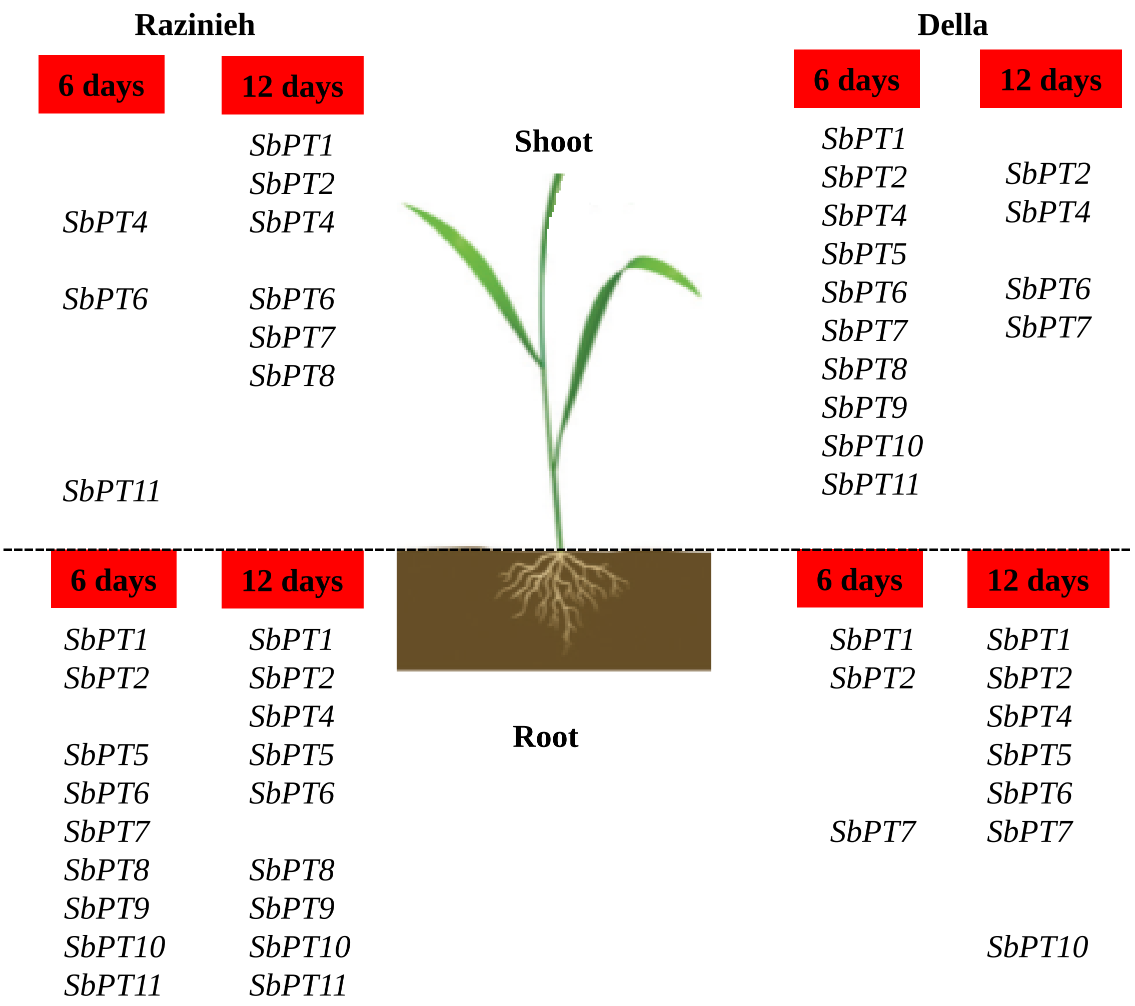

Figure S2. Schematic models of the upregulated *SbPht1* genes family in leaves and roots of a grain (Razinieh) and a sweet (Della) sorghum varieties grown under P_i starvation treatments. The seeds of two cultivars were initially grown in Agar with 8% MS for 10 days, after that the seedlings were transferred to half Hoagland solution for 2 days, followed by full Hoagland solution for 4 days to get more adaptation. Thereafter, the seedling were subjected to 2mM $NH_4H_2PO_4$ (normal condition; $+P_i$) or zero (phosphorous starvation; $-P_i$). After that, plant sampling for RNA extraction was done at 0, 6 and 12 days of P_i starvation. Transcript levels were normalized against the ubiquitin housekeeping gene. Values are means of three biological replicates. Raz, Razinieh. d, day. The color scale is shown on the right side. Heat map of gene expression profiles was generated using GraphPad Prism 8.4.3 software after data normalization.

Table S1: Physical and chemical properties of soil.

Characters	Soil with normal phosphate content	Soil with low phosphate content
Soil type	Slightly loamy sand	Silty clay
pH value	6.2	7.4
Potassium (K_2O)	9 mg / 100g	7 mg / 100g
Magnesium (Mg)	8 mg / 100g	10 mg / 100g
Phosphorus (P_2O_5)	19 mg / 100g (fertilized to 40 mg / 100g with calcium dihydrogen phosphate)	4 mg / 100g
Total N (Dumas)	0.09%	0.24%
Humus	1.9%	4.1%
water capacity	65%	65%

Table S2: Chemical composition of modified Hoagland's solution.

Compounds	Molecular weight	Concentration of stock solution	Concentration of stock solution	Volume of stock solution per liter of final solution	Elements	Final concentration of elements	ppm
Macronutrients	g mol^{-1}	mM	gL^{-1}	ml		uM	
KNO_3	101.1	1	101.1	6	N	16000	224
$Ca(NO)_3 * 4H_2O$	236.16	1	236.16	4	K	6000	235
$NH_4H_2PO_4$	115.08	1	115.08	2	Ca	4000	160
$MgSO_4 * 7H_2O$	246.48	1	246.49	1	P	2000	62
					S	1000	32
					Mg	1000	24
Micronutrients							
KCl	74.55	25	1.864		Cl	50	1.77
H_2BO_3	61.83	12.5	0.773		B	25	0.27
$MnSO_4 * H_2O$	169.01	1	0.169		Mn	2	0.11
$ZnSO_4 * 7H_2O$	287.54	1	0.288	2	Zn	2	0.13
$CuSO_4 * 5H_2O$	249.68	0.25	0.062		Cu	0.5	0.03
H_2MoO_4 (85% MoO_3)	161.97	0.25	0.04		Mo	0.5	0.05
NaFeDTPA(10% Fe)	468.2	64	30	0,3-1	Fe	16,1-53,7	1,0-3,0

Table S3: Primers for qRT PCR analysis of *Pht1* transcripts of sorghum.

Name	Sequence (5'-3')
SbPT1-f	GGCCAAGGTGCTCAAGAAG
SbPT1-r	GGAGGAACTGCACCGAGAAG
SbPT2-f	ACTAAGCAGCAGCCTCCGTA
SbPT2-r	AAGCCACAAGGAAACCATTG
SbPT4-f	GGCGCCGTCGTACCAGGACAA
SbPT4-r	GAGCGCCGCCGGGATGGT
SbPT5-f	GAGAATCTGGACGAGATCAC
SbPT5-r	CAGGTTCTGGCTGTAGTAGG
SbPT6-f	CAAGCTCGGCCGTAAGAAGG
SbPT6-r	GCCAGAAGCGGAAGAAGCAC
SbPT7-f	GGACACCAGCAAGGACAAC
SbPT7-r	CGCGATGGAGCAGATGAC
SbPT8-f	GCAGCGAGGCCAATGAGACT
SbPT8-r	TTGGCTCCGGTAGGAAGCAG
SbPT9-f	GAGGACGAGCCGTTCAAGAG
SbPT9-r	CGCGACGGAGAAGAAGTACC
SbPT10-f	CACCATGTGCTGGTTACTTC
SbPT10-r	GATAATCGCCTGAGTACGTG
SbPT11-f	CGTGGTTCCTTCTGGACATA
SbPT11-r	TCTCGAACACCTCCTTGAGT
SbUBI-f	CAAGGAGTGCCCCAACAC
SbUBI-r	TGGTAGGCGGGTAAAGCAAA
